# Endogenous PTH Deficiency Impairs Fracture Healing and Impedes the Fracture-Healing Efficacy of Exogenous PTH(1-34)

**DOI:** 10.1371/journal.pone.0023060

**Published:** 2011-07-29

**Authors:** Yongxin Ren, Bo Liu, Yuxu Feng, Lei Shu, Xiaojian Cao, Andrew Karaplis, David Goltzman, Dengshun Miao

**Affiliations:** 1 Department of Orthopaedics, The First Affiliated Hospital, Nanjing Medical University, Nanjing, China; 2 Department of Orthopaedics, The Liyang People's Hospital, Changzhou, China; 3 The Research Center for Bone and Stem Cells, Department of Anatomy, Histology and Embryology, Nanjing Medical University, Nanjing, China; 4 Department of Medicine, McGill University, Montreal, Canada; University of Western Ontario, Canada

## Abstract

**Background:**

Although the capacity of exogenous PTH1-34 to enhance the rate of bone repair is well established in animal models, our understanding of the mechanism(s) whereby PTH induces an anabolic response during skeletal repair remains limited. Furthermore it is unknown whether endogenous PTH is required for fracture healing and how the absence of endogenous PTH would influence the fracture-healing capacity of exogenous PTH.

**Methodology/Principal Findings:**

Closed mid-diaphyseal femur fractures were created and stabilized with an intramedullary pin in 8-week-old wild-type and *Pth* null (*Pth* ^−/−^) mice. Mice received daily injections of vehicle or of PTH1-34 (80 µg/kg) for 1–4 weeks post-fracture, and callus tissue properties were analyzed at 1, 2 and 4 weeks post-fracture. Cartilaginous callus areas were reduced at 1 week post-fracture, but were increased at 2 weeks post-fracture in vehicle-treated and PTH-treated *Pth* ^−/−^ mice compared to vehicle-treated and PTH-treated wild-type mice respectively. The mineralized callus areas, bony callus areas, osteoblast number and activity, osteoclast number and surface in callus tissues were all reduced in vehicle-treated and PTH-treated *Pth* ^−/−^ mice compared to vehicle-treated and PTH-treated wild-type mice, but were increased in PTH-treated wild-type and *Pth* ^−/−^ mice compared to vehicle-treated wild-type and *Pth* ^−/−^ mice.

**Conclusions/Significance:**

Absence of endogenous PTH1-84 impedes bone fracture healing. Exogenous PTH1-34 can act in the absence of endogenous PTH but callus formation, including accelerated endochondral bone formation and callus remodeling as well as mechanical strength of the bone are greater when endogenous PTH is present. Results of this study suggest a complementary role for endogenous PTH1-84 and exogenous PTH1-34 in accelerating fracture healing.

## Introduction

The major form of bioactive parathyroid hormone (PTH) secreted by the parathyroid glands is an 84-amino acid peptide. In response to a hypocalcemic stimulus increased endogenous PTH release can raise extracellular calcium levels by indirectly enhancing gastrointestinal calcium absorption through its action on stimulating 1,25 dihydroxyvitamin D (1,25(OH)_2_D) production, and by directly enhancing renal calcium reabsorption and osteoclastic bone resorption, thereby releasing calcium from the skeleton. Although endogenous PTH1-84, is classically believed to function as a bone resorbing hormone to maintain calcium homeostasis, endogenous PTH also has a critical role to play in the development and maintenance of trabecular bone mass, particularly in the fetus and the neonate. We previous demonstrated this skeletal anabolic role for endogenous PTH1-84 using a genetic approach [1], [2]. We found that osteoblast number and trabecular bone volume were reduced in newborn [1] and 2-week-old [2] PTH null mice. We have previously reported a mouse model deficient in 1,25(OH)_2_D by targeted ablation of the 25-hydroxyvitamin D-1α-hydroxylase enzyme (1α(OH)ase^−/−^) [3]. After weaning, mice fed a diet of regular mouse chow developed hyperparathyroidism, and exhibited the retarded growth and skeletal abnormalities characteristic of rickets. Bone volume and osteoblast numbers were increased in the 1α(OH)ase^−/−^ animals with secondary hyperparathyroidism [3]. These increases most likely also reflect “anabolic” activity of endogenous PTH1-84.

Numerous studies have demonstrated that exogenous PTH1-84, and exogenous PTH1-34 (a synthetic polypeptide that comprises the bioactive 1–34 amino acid fragment of PTH), when administered intermittently rather than continuously, can augment bone mass [4], [5] and both PTH1-84 and PTH1-34, either of which can bind to the type I PTH/PTHrP receptor (PTHR1), are in clinical use for the treatment of osteoporosis. When injected subcutaneously once a day in osteoporotic patients, it stimulates cancellous bone formation, increases bone mineral density (BMD), and reduces the risk of fracture [6]. The capacity of daily injections of exogenous PTH1-34 [7], [8], [9], [10], [11] or exogenous PTH1-84 [12] to enhance fracture healing has been examined in several rodent models. All showed significant increases in the mechanical properties of the calluses, rates of callus formation, and callus tissue volume [7], [8], [9], [11]. In other studies using models of impaired bone metabolism, PTH analogs or PTH were shown to reverse the inhibition of bone healing observed in aged and ovariectomized rats [10], [12]. Although the ability of exogenous PTH to enhance the rate of bone repair is thus well established in animal models, it is unknown whether endogenous PTH1-84 is required for fracture healing.

To assess whether endogenous PTH1-84 deficiency impairs fracture healing and to determine what the effect of the absence of endogenous PTH1-84 might be on the capacity of exogenous PTH1-34 to accelerate fracture healing, closed mid-diaphyseal femur fractures were created and stabilized with an intramedullary pin in 8-week-old wild-type and *Pth*-null (*Pth* ^−/−^) mice. Mice received daily injections of vehicle or of PTH1-34 (80 µg/kg) for 1–4 weeks post-fracture, and callus tissue properties was analyzed at 1, 2 and 4 weeks post-fracture by radiography, micro-CT, histology, histochemistry and immunohistochemistry. RNA and proteins were isolated from callus tissues and bone formation-related gene and protein expression levels were evaluated by real-time RT-PCR and Western blots, respectively.

## Materials and Methods

### Derivation of *Pth* null mice

The derivation of the parental strain of *Pth^−/−^* mice by homologous recombination in embryonic stem cells and genotyping of mice were previously described by Miao et al [1], [13]. Briefly, a neomycin resistance gene was inserted into exon III of the mouse *Pth* gene replacing the entire mature *Pth* coding sequence. Lack of PTH expression was confirmed by immunostaining of parathyroid gland sections [1]. Eight-week-old wild-type and *Pth* ^−/−^ mice were used in this study.

### Creation of femur fractures and administration of PTH1-34

A standardized mid-diaphyseal fracture was induced in 36 8-week-old wild-type mice and 36 8-week-old *Pth* ^−/−^ mice as described previously [14]. Briefly, after sedation of the animals with isoflurane (Forene®) and intraperitoneal anesthesia with a mixture of ketamine hydrochloride (100 mg/ml; 80 mg/kg body weight) and xylazin 2% (12 mg/kg body weight), the right hind leg was shaved and disinfected. An intramedullary pin (Ø = 0.5 mm) was introduced into the femoral canal through a medial parapatellar incision and arthrotomy of the knee. After closing the wound, a mid-diaphyseal fracture was produced by using a falling weight of 300 g over a threepoint bending mechanism. In all groups, the drop height of the weight was 15 cm. The fracture was radiographically documented. In all cases, a straight mid-diaphyseal fracture was induced. At the time of fracture, wild type and *Pth^−/−^* mice received daily injections of vehicle or of PTH1-34 (80 µg/kg) subcutaneously for 1–4 weeks. The use of animals in this study was approved by the Institutional Animal Care and Use Committee of Nanjing Medical University (Approval ID 2008-00318).

### Skeletal radiography

Femurs were removed and dissected free of soft tissue from 6 mice of each group. Contact radiographs were taken using a Faxitron model 805 radiographic inspection system (Faxitron Contact, Faxitron, Germany) (22 kV voltage and 4 min exposure time). X-Omat TL film (Eastman Kodak Co., Rochester, NY, USA) was used and processed routinely.

### Micro-computed tomography

After radiography examination, femurs were analyzed by micro-CT with a SkyScan 1072 scanner and associated analysis software (SkyScan, Antwerp, Belgium) as described [2]. Briefly, image acquisition was performed at 100 kV and 98 mA with a 0.98 rotation between frames. During scanning, the samples were enclosed in tightly fitting plastic wrap to prevent movement and dehydration. Thresholding was applied to the images to segment the bone from the background. Two-dimensional images were used to generate three-dimensional renderings using the 3D Creator software supplied with the instrument. The resolution of the micro-CT images is 18.2 µm. A volume of interest for quantitative analysis was defined, extending from the proximal to the distal end of the callus region. For each 3D image, total bone volume was calculated, including both the cortical bone and the surrounding new bone formation. Following manual segmentation, a second evaluation was then performed to calculate bone volume of the cortical bone alone. Finally, the calcified callus volume was determined by subtracting the cortical bone volume from the total volume.

### Biomechanical testing

Four weeks after fracture, femurs of 6 vehicle-treated wild-type and *Pth* ^−/−^ mice and 6 PTH-treated wild-type and *Pth* ^−/−^ mice were rehydrated at room temperature in phosphate buffered saline (PBS) and their biomechanical properties were assessed by a three-point bending test. Strength tests were performed at the right femur midshaft with a displacement rate of 10 mm/minute (span length, 10 mm) using a mechanical testing machine (model 5865; Instron, Norwood, MA). Whole-bone mechanical properties, including maximum load, maximum stress and energy-to-failure, were determined using load-deflection diagrams. Maximum stress was calculated as σ  =  y F L/4 I, where σ  =  maximum stress (Pa (N/m^2^), N/mm^2^, psi), y  =  Perpendicular distance from to neutral axis (m, mm, in), F  =  load (N, lb), L  =  length between two supports (m, mm, in), I  =  moment of Inertia (m^4^,mm^4^, in^4^).

### Western blot analysis

Proteins were extracted from 6 callus tissues of each group and quantitated using a kit (Bio-Rad, Mississauga, Ontario, Canada). Thirty-microgram protein samples were fractionated by SDS-PAGE and transferred to nitrocellulose membranes. Immunoblotting was carried out as described [15] using antibodies against insulin-like growth factor 1 (IGF-1) (Santa Cruz, CA, USA) and core binding factor alpha1 (Cbfa1) (Santa Cruz, CA, USA) and β-tubulin (Santa Cruz, CA, USA) was used as a loading control. Bands were visualized using enhanced chemiluminescence (Amersham, Aylesbury, UK).

### Quantitative real-time RT-PCR

RNA was isolated from 6 callus tissues of each group, using Trizol reagent (Invitrogen) according to the manufacturer's protocol. Reverse transcription reactions were performed using the SuperScript First-Strand Synthesis System (Invitrogen) as previously described [2]. To determine the number of cDNA molecules in the reverse transcribed samples, real-time PCR analyses were performed using the LightCycler system (Roche, Indianapolis, IN). PCR was performed using 2 µl LightCycler DNA Master SYBR Green I (Roche), 0.25 µM of each 5′ and 3′ primer, and 2 µl samples or H_2_O to a final volume of 20 µl. The MgCl_2_ concentration was adjusted to 3 mM. Samples were denatured at 95°C for 10 sec, with a temperature transition rate of 20°C per sec. Amplification and fluorescence determination were carried out in four steps: denaturation at 95°C for 10 sec, with a temperature transition rate of 20°C/sec; annealing for 5 sec, with a temperature transition rate of 8°C/sec; extension at 72°C for 20 sec, with a temperature transition rate of 4°C/sec; and detection of SYBR Green fluorescence, which reflects the amount of double-stranded DNA, at 86°C for 3 sec. The amplification cycle number was 35. To discriminate specific from nonspecific cDNA products, a melting curve was obtained at the end of each run. Products were denatured at 95°C for 3 sec, and the temperature was then decreased to 58°C for 15 sec and raised slowly from 58 to 95°C using a temperature transition rate of 0.1°C/sec. To determine the number of copies of the targeted DNA in the samples, purified PCR fragments of known concentrations were serially diluted and served as external standards that were measured in each experiment. Data were normalized with GAPDH levels in the samples. The primer sequences used for the real-time PCR were the same as described previously [16].

### Histology

Femurs were removed from 6 mice of each group and fixed in PLP fixative (2% paraformaldehyde containing 0.075 M lysine and 0.01 M sodium periodate) overnight at 4°C and processed histologically as described [17]. Femurs were decalcified in ethylene-diamine tetraacetic acid (EDTA)-glycerol solution for 5–7 days at 4°C. Decalcified femurs were dehydrated and embedded in paraffin, after which 5 µm sections were cut on a rotary microtome. The sections were stained with hematoxylin and eosin (HE) or histochemically for total collagen and alkaline phosphatase (ALP) activity and tartrate-resistant acid phosphatase (TRAP) or immunohistochemical staining as described below.

### Histochemical staining for collagen, ALP and TRAP

Total collagen was detected in paraffin-embedded sections using a modification of the method of Lopez-De Leon and Rojkind as described previously [18]. Dewaxed sections were exposed to 1% sirius red in saturated picric acid for 1 h. After washing with distilled water, the sections were counterstained with hematoxylin and mounted with Biomount medium.

Enzyme histochemistry for ALP activity was performed as described in paraffin sections [19]. Briefly, following preincubation overnight in 1% magnesium chloride in 100 mM Tris–maleate buffer (pH 9.2), de-waxed sections were incubated for 2 h at room temperature in a 100 mM Tris–maleate buffer containing naphthol AS-MX phosphate (0.2 mg/ml, Sigma) dissolved in ethylene glycol monomethyl ether (Sigma-Aldrich, St Louis, MO, USA) as substrate and fast red TR (0.4 mg/ml, Sigma) as a stain for the reaction product. After washing with distilled water, the sections were counterstained with Vector methyl green nuclear counterstain (Vector Laboratories, Burlingame, CA, USA) and mounted with Kaiser's glycerol jelly.

Enzyme histochemistry for TRAP was performed on paraffin sections using a modification of a previously described protocol [20]. Dewaxed sections were preincubated for 20 min in buffer containing 50 mM sodium acetate and 40 mM sodium tartrate at pH 5.0. Sections were then incubated for 15 min at room temperature in the same buffer containing 2.5 mg/ml naphthol AS-MX phosphate (Sigma) in dimethylformamide as substrate and 0.5 mg/ml fast garnet GBC (Sigma) as a color indicator for the reaction product. After washing with distilled water, the sections were counterstained with methyl green and mounted in Kaiser's glycerol jelly.

### Immunohistochemical staining

Immunohistochemical staining for type I collagen was performed on paraffin sections using the avidin-biotin-peroxidase complex technique with affinity-purified goat anti-human type I collagen antibody (Southern Biotechnology Associates, Birmingham, AL, USA). Briefly, dewaxed and rehydrated paraffin-embedded sections were incubated with methanol-hydrogen peroxide (1∶10) to block endogenous peroxidase activity and then washed in Tris-buffered saline (pH 7.6). The slides were then incubated with the primary antibody overnight at room temperature. After rinsing with Tris-buffered saline for 15 min, tissues were incubated with biotinylated secondary antibody (Sigma). Sections were then washed and incubated with the Vectastain Elite ABC reagent (Vector Laboratories) for 45 min. After washing, brown pigmentation was likewise produced using 3,3-diaminobenzidine (2.5 mg/ml). After washing with distilled water, the sections were counterstained with Mayer's hematoxylin, dehydrated in graded ethanol and xylene and mounted with Biomount medium.

### Computer-assisted image analysis

After HE staining, or histochemical or immunohistochemical staining of sections from six mice of each genotype, images of fields were photographed with a Sony digital camera. Images of micrographs from single sections were digitally recorded using a rectangular template, and recordings were processed and analyzed using Northern Eclipse image analysis software as described [2], [17]. All measurements were performed in a blinded fashion. The region of interest (ROI) was defined as the area of the callus on both sides of the medullary canal excluding all cortical bone. This ROI was outlined manually for each specimen using the software.

### Statistical Analysis

Data from image analysis are presented as mean ± SEM. Statistical comparisons were made using a two-way ANOVA, with P<0.05 being considered significant.

## Results

### Effects of endogenous PTH deficiency and of exogenous PTH on bone fracture healing

**Figure 1:**
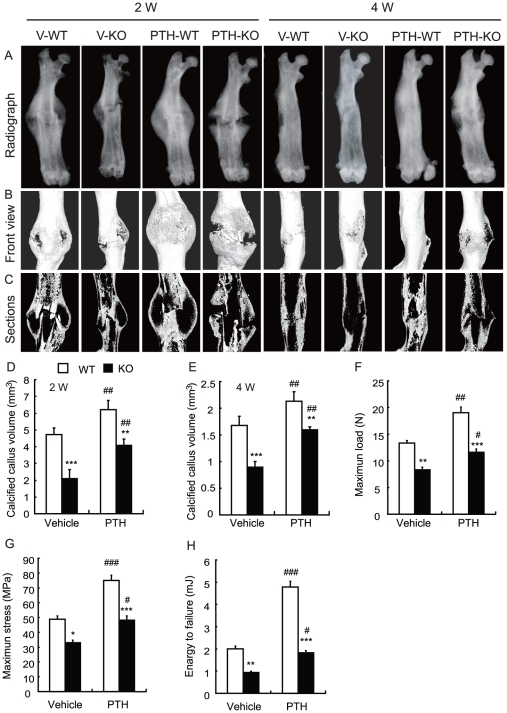
Effects of endogenous PTH deficiency and exogenous PTH on bone fracture healing and mechanical properties of fractured femurs. (A) Representative radiographs of femurs from vehicle-treated wild-type (V-WT) and vehicle-treated *Pth* ^−/−^ (V-KO) mice and PTH-treated wild-type (PTH-WT) and PTH-treated *Pth* ^−/−^ (PTH-KO) mice at 2 weeks (2W) and 4 weeks (4W) post-fracture. Representative (B) frontal views and (C) longitudinal sections of micro-CT 3-dimensional reconstructed calluses from V-WT and V-KO mice and PTH-WT and PTH-KO mice at 2 week and 4 weeks post-fracture. The quantitative analysis for calcified callus volume at (D) 2 weeks (2W) and (E) 4 weeks (4W) post-fracture. (F) Maximum load, (G) maximum stress and (H) energy-to failure were assessed by three-point bending in femurs from 4 weeks post-fracture in vehicle-treated wild-type (WT) and *Pth* ^−/−^ mice (KO) and PTH-treated wild-type and *Pth* ^−/−^ mice. Each value is the mean ± SEM of determinations in 6 animals from each group. *, P<0.05; **, P<0.01; ***, P<0.001 compared with WT mice at the same group. #, P<0.05; ##, P<0.01; ###, P<0.001 compared with genotype-matched V-treated mice.

Effects of endogenous PTH1-84 deficiency and exogenous PTH1-34 on bone fracture healing were examined at 2 and 4 weeks post-fracture in vehicle-treated wild-type and *Pth* ^−/−^ mice and in PTH-treated wild-type and *Pth* ^−/−^ mice by radiography (Fig. 1A) and micro-CT (Figs. 1B and C). By radiography (Fig. 1A), the front view (Fig. 1B) and longitudinal sections (Fig. 1C) of micro-CT 3-dimensional reconstructions and quantitative analysis (Figs. 1D and E) demonstrated that the size of callus and the calcified callus volume at 2 weeks post-fracture were reduced in vehicle-treated and PTH-treated *Pth* ^−/−^ mice compared to vehicle-treated and PTH-treated wild-type mice, respectively, and were increased in PTH-treated wild-type and *Pth* ^−/−^ mice compared to vehicle-treated wild-type and PTH-treated *Pth* ^−/−^ mice respectively. In addition, the fracture repairs at 4 weeks post-fracture were poor in *Pth* ^−/−^ mice compared to wild-type mice, and were improved significantly in PTH1-34-treated wild-type and *Pth* ^−/−^ mice. However, the best fracture repairs were observed in the PTH-treated wild-type mice.

### Effects of endogenous PTH deficiency and of exogenous PTH on mechanical properties of fractured femurs at 4 weeks post-fracture

To assess effects of endogenous PTH deficiency and of exogenous PTH on mechanical properties of fractured femurs, biomechanical properties were examined by three-point bending in femurs 4 weeks after fracture in vehicle-treated wild-type and *Pth* ^−/−^ mice and PTH-treated wild-type and *Pth* ^−/−^ mice. Results revealed that maximum load (Fig. 1F), maximum stress (Fig. 1G) and energy-to failure (Fig. 1H) were all reduced in vehicle-treated and PTH-treated *Pth^−/−^* mice compared to vehicle-treated and PTH-treated wild-type mice, respectively, and were increased in PTH-treated wild-type and *Pth* ^−/−^ mice compared to vehicle-treated wild-type and *Pth* ^−/−^ mice, respectively.

### Effects of endogenous PTH deficiency and of exogenous PTH on cartilaginous callus formation and on the transformation into bony callus

**Figure 2:**
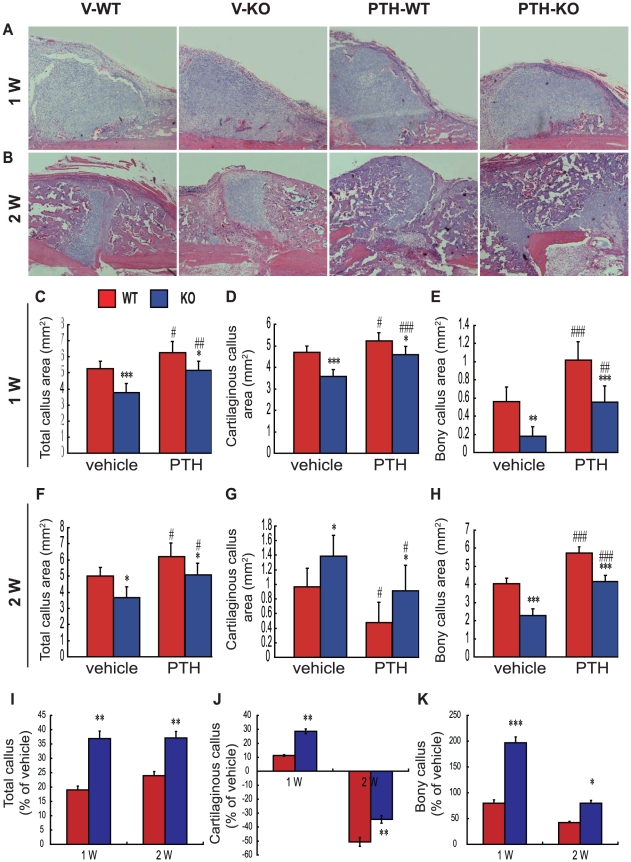
Effects of endogenous PTH deficiency and exogenous PTH on cartilaginous callus formation and on the transformation into bony callus. Representative micrographs of paraffin sections of calluses from V-WT and V-KO mice, and PTH-WT and PTH-KO mice at (A) 1 week and (B) 2 weeks post-fracture. Areas of the total callus (C, F), cartilaginous callus (D, G) and bony callus (E, H) were measured by computer-assisted image analysis. The percentage of increased or reduced (I) total callus, (J) cartilaginous callus and bony callus (K) in response to PTH at 1 and 2 weeks post-fracture in wild type (WT) and *Pth* ^−/−^ (KO) mice. Each value is the mean ± SEM of determinations in 6 animals from each group. *, P<0.05; **, P<0.01; ***, P<0.001 compared with WT mice at the same group. #, P<0.05; ##, P<0.01; ###, P<0.001 compared with genotype-matched V-treated mice.

To determine whether endogenous PTH deficiency and exogenous PTH affect cartilaginous callus formation and its transformation into bony callus, callus tissues from vehicle-treated wild-type and *Pth* ^−/−^ mice and PTH-treated wild-type and *Pth* ^−/−^ mice were analyzed at 1 and 2 weeks post-fracture by histology and computer-assisted image analysis. Results showed that at 1 week post-fracture, total callus areas, cartilaginous callus areas and bony callus areas were reduced in vehicle-treated and PTH-treated *Pth* ^−/−^ mice compared to vehicle-treated and PTH-treated wild-type mice respectively, but were increased in both PTH-treated wild-type and *Pth* ^−/−^ mice compared to vehicle-treated wild-type and *Pth* ^−/−^ mice respectively (Figs. 2A, C–E). At 2 weeks post-fracture, total callus areas and bony callus areas were reduced in vehicle-treated and PTH-treated *Pth* ^−/−^ mice compared to vehicle-treated and PTH-treated wild-type mice respectively, but were increased in both PTH-treated wild-type and *Pth* ^−/−^ mice compared to vehicle-treated wild-type and *Pth* ^−/−^ mice respectively (Figs. 2B, F and H). In contrast, at 2 weeks post-fracture, remnant cartilaginous callus areas were increased in vehicle-treated and PTH-treated *Pth* ^−/−^ mice compared to vehicle-treated and PTH-treated wild-type mice respectively, and were reduced in PTH-treated wild-type and *Pth* ^−/−^ mice compared to vehicle-treated wild-type and *Pth* ^−/−^ mice, respectively (Figs. 2B and G). The percentage of increased total callus and bony callus in response to PTH was greater at 1 and 2 weeks post-fracture in *Pth* ^−/−^ mice than in wild type mice (Figs. 2I and K). The percentage of increased cartilaginous callus in response to PTH was greater at 1 week post-fracture in *Pth* ^−/−^ mice than in wild type mice, whereas the percentage of reduced cartilaginous callus in response to PTH was less at 2 week post-fracture in *Pth* ^−/−^ mice than in wild type mice (Fig. 2J).

### Effects of endogenous PTH deficiency and of exogenous PTH on the expression of osteoblastic bone formation related genes and proteins in callus tissues at 1 and 2 weeks post-fracture

**Figure 3:**
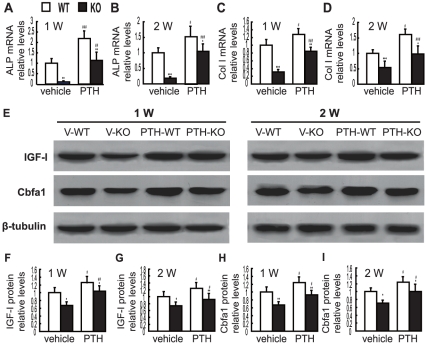
Effects of endogenous PTH deficiency and exogenous PTH on the expression of osteoblastic bone formation related genes and proteins in callus tissues at 1 and 2 weeks post-fracture. Real-time RT–PCR was performed on callus extracts from V-WT and V-KO mice and PTH-WT and PTH-KO mice at 1 week and 2 weeks post-fracture for gene expression of (A, B) ALP and (C, D) type I collagen (Col I). Messenger RNA expression was calculated as a ratio relative to the GAPDH mRNA level and expressed relative to levels of V-WT mice. (E) Western blots for protein expression of Cbfa1, and insulin-like growth factor-1 (IGF-1), of callus extracts from V-WT and V-KO mice and PTH-WT and PTH-KO mice at 1 week and 2 weeks post-fracture. β-tubulin was used as loading control for Western blots. (F, G) Cbfa1 and (H, I) IGF-1 protein levels relative to β-tubulin protein level were assessed by densitometric analysis and expressed relative to levels of V-WT mice. Each value is the mean ± SEM of determinations in 6 animals from each group. *, P<0.05; **, P<0.01; ***, P<0.001 compared with WT mice of the same group. #, P<0.05; ##, P<0.01; ###, P<0.001 compared with genotype-matched V-treated mice.

To determine whether the alterations of the bony callus areas were associated with the regulation of the expression of osteoblastic bone formation related genes and proteins in callus tissues, mRNA and proteins were isolated from callus extracts from vehicle-treated wild-type and *Pth* ^−/−^ mice and PTH-treated wild-type and *Pth* ^−/−^ mice. The expression of ALP and type I collagen genes and the expression of Cbfa1 and IGF-1 proteins were examined at 1 and 2 weeks post-fracture by real-time RT–PCR and Western blots, respectively. At 1 and 2 weeks post-fracture, the mRNA levels of ALP and type I collagen, and the protein levels of Cbfa1 and IGF-1 were reduced in vehicle-treated and PTH-treated *Pth* ^−/−^ mice compared to vehicle-treated and PTH-treated wild-type mice, respectively, and were increased in PTH-treated wild-type and *Pth* ^−/−^ mice compared to vehicle-treated wild-type and PTH-treated *Pth* ^−/−^ mice, respectively (Fig. 3).

### Effects of endogenous PTH deficiency and of exogenous PTH on osteoblastic bone formation in calluses at 4 weeks post-fracture

**Figure 4:**
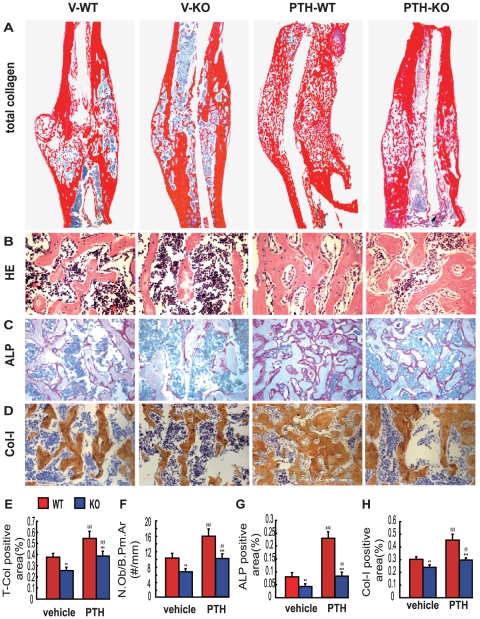
Effects of endogenous PTH deficiency and exogenous PTH on osteoblastic bone formation in calluses at 4 weeks post-fracture. Representative micrographs of paraffin sections of calluses from V-WT and V-KO mice and PTH-WT and PTH-KO mice at 4 weeks post-fracture stained with (A) Sirius Red for total collagen, (B) hematoxylin and eosin (HE), (C) histochemically for ALP and (D) immunohistochemically for type I collagen (Col I). (E) Total collagen positive bony callus areas, (F) osteoblast number relative to bone perimeter (N.Ob/B.Pm, #/mm), (G) ALP positive areas and (H) Col I immunopositive areas were measured by computer-assisted image analysis. Each value is the mean ± SEM of determinations in 6 animals from each group. **, P<0.01; ***, P<0.001 compared with WT mice of the same group. #, P<0.05; ##, P<0.01; ###, P<0.001 compared with genotype-matched V-treated mice.

To determine effects of endogenous PTH deficiency and exogenous PTH on osteoblastic bone formation in calluses, bony callus volume and osteoblast number and activity were examined at 4 weeks post-fracture by histology, histochemistry and immunohistochemistry. At 4 weeks post-fracture, total collagen positive bony callus areas, osteoblast number, ALP positive areas and type I collagen immunopositive areas were all reduced in vehicle-treated and PTH-treated *Pth^−/−^* mice compared to vehicle-treated and PTH-treated wild-type mice, respectively, and were increased in PTH-treated wild-type and *Pth* ^−/−^ mice compared to vehicle-treated wild-type and *Pth* ^−/−^ mice, respectively (Fig. 4).

### Effects of endogenous PTH deficiency and of exogenous PTH on osteoclastic bone resorption in calluses at 2 and 4 weeks post-fracture

**Figure 5:**
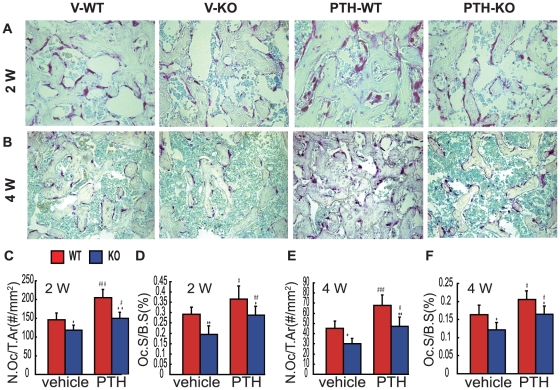
Effects of endogenous PTH deficiency and exogenous PTH on osteoclastic bone resorbtion in calluses at 2 and 4 weeks post-fracture. Representative micrographs of sections of calluses from V-WT and V-KO mice and PTH-WT and PTH-KO mice at (A) 2 weeks and (B) 4 weeks post-fracture stained histochemically for TRAP. (C, D) Number of TRAP-positive osteoclasts related to tissue area (N.Oc/T.Ar, #/mm2) and (E, F) osteoclast surface relative to bone surface (Oc.S/BS, %) were assessed by computer-assisted image analysis. Each value is the mean ± SEM of determinations in 6 animals from each group. *, P<0.05; **, P<0.01 compared with WT mice of the same group. #, P<0.05; ##, P<0.01; ###, P<0.001 compared with genotype-matched V-treated mice.

To determine whether endogenous PTH deficiency and exogenous PTH affect osteoclastic bone resorption during bone fracture healing, osteoclastic bone resorption was examined in callus tissues at 2 and 4 weeks post-fracture by histochemical staining and computer-assist image analysis. Results revealed that at 2 and 4 weeks post-fracture, the TRAP positive osteoclast number and surface were reduced in vehicle-treated and PTH-treated *Pth* ^−/−^ mice compared to vehicle-treated and PTH-treated wild-type mice respectively,but were increased in PTH-treated wild-type and *Pth* ^−/−^ mice compared to vehicle-treated wild-type and *Pth* ^−/−^ mice respectively (Fig. 5).

## Discussion

Our study demonstrates that deficiency of endogenous PTH impairs the fracture repair process, by reducing cartilaginous and bony callus formation with down-regulation of osteoblastic gene and protein expression, and reduction of endochondral bone formation, osteoblastic bone formation and osteoclastic bone resorption. These results indicate that endogenous PTH plays an important role in fracture healing. These findings are consistent with a previous study that reported that in parathyroidectomized rats with decreased serum PTH, fracture healing was impaired due to delay of both chondroclasts at the phase of endochondral ossification and secondary remodeling of primary cancellous bone [21]. We previously reported that *Pth* ^−/−^ mice which have absent endogenous PTH, have decreased osteoblast and osteoclast numbers and diminished trabecular bone volume at birth, demonstrating that PTH is essential for fetal trabecular bone formation [1]. We also observed similar phenotypic alterations in 2-week-old *Pth* ^−/−^ mice in that trabecular bone formation and trabecular bone volume were reduced when compared with wild-type littermates [2]. Consequently endogenous PTH appears to play an important role be in the regulation of normal trabecular bone formation. *Pth* ^−/−^ mice also displayed reduced mineralization and diminished vascular invasion at the chondro-osseous junction at birth [1] and 2 weeks of age [2]. This important role for endogenous PTH in endochondral bone formation during normal development may therefore be recapitulated in the process of endochondral bone formation occurring during the fracture healing process.

Several animal studies have documented that exogenous PTH, either PTH1-34 or PTH1-84, enhances fracture healing and is able to stimulate hard-callus formation and increase the strength of the fracture site. Our results also demonstrated that PTH1-34 treatment of wild type mice enlarged the cartilaginous callus and accelerated cartilaginous callus transformation into bony callus. Upregulation of IGF-1 was observed in our studies in response to exogenous PTH treatment. It has previously been suggested that IGF-I mediates the proliferative effect of PTH on mesenchymal cells, which may then differentiate into cells of the chondrogenic lineage, thereby contributing to the increased size of the cartilaginous callus upon PTH1–34 treatment [22]. Our studies are consistent with those observations. Studies have also suggested direct effects of PTH on chondrocyte proliferation and differentiation [22], [23]. We previously reported that the proliferation and apoptosis of chondrocytes were not altered significantly in newborn and 2-week-old *Pth* ^−/−^ mice which lack endogenous PTH. Indeed PTH, which is not expressed in chondrocytes or cartilaginous callus, and which must access PTHR1 via the blood stream (whether it is of endogenous or exogenous origin), is unlikely to directly interact with chondrocytes in the avascular cartilaginous growth plate [1], [2]. It is however possible that chondrocyte proliferation and differentiation are influenced indirectly by increases in extracellular calcium observed after exogenous PTH1-34 administration. The increase in bony callous formation was accompanied by up-regulation of Cbfa1 protein levels and by up-regulation of ALP and type I collagen mRNA levels and at 1 and 2 weeks post-fracture, by increased bony callus volume, osteoblast number, ALP and type I collagen positive areas at 4 weeks post-fracture, and by increased osteoclast number and surface at 2 and 4 weeks post-fracture. Exogenous PTH1-34 therefore not only stimulated bony callus formation by enhancing osteoblastic bone formation but also accelerated bony callus remodeling by stimulating osteoclastic bone resorption. Taken together these data indicate that exogenous PTH 1–34 promotes fracture healing by stimulating proliferation of osteoblastic progenitor cells, synthesis of bone matrix proteins, and osteoclastogenesis, thereby enhancing both callus formation and callus remodeling. The resulting bones after 4 weeks of treatment with PTH showed better mechanical strength than fractured bones of wild type mice treated with vehicle only.

We also found that the administration of exogenous PTH1-34 rescued impaired fracture healing in mice deficient in endogenous PTH. Interestingly the percent increase in total callous and in bony callous and in indices of bone formation were generally higher in the PTH1-34-treated *Pth* ^−/−^ mice than in the PTH1-34-treated wild type mice. Increases in renal phosphaturic and cyclic AMP responses to exogenous PTH have previously been reported in hypoparathyroid patients [24]; the apparent increased responsiveness to exogenous PTH1-34 in fracture healing may therefore be evidence of similar enhanced sensitivity in bone. Whether this apparent relative increase in responsiveness is secondary to upregulation of PTHR1 in the absence of PTH ligand in hypoparathyroidism or to some other mechanism will require further study. Nevertheless despite these relative increases, using the same does of exogenous PTH1-34, the final amount of total callous and bony callous were highest in the PTH-treated wild type mice which produce endogenous PTH, and this higher volume of bony callous was associated with bones exhibiting the most favorable mechanical properties.

Intermittent administration of PTH1-34 or PTH 1-84 has an “anabolic” effect on bone although primarily on trabecular bone and not cortical bone. Although endogenous PTH under physiologic conditions is presumably secreted relatively continuously we have previously shown it to be essential for trabecular bone formation in the fetus and neonate [1], [2]. Secondary hyperparathyroidism, where continuous PTH secretion does occur, is also associated with increased osteoblast activity and increased bone matrix production [3]. Primary hyperparathyroidism and continuous administration of exogenous PTH, whether 1–84 or its active fragment 1–34, are known to produce bone resorption although primarily in cortical bone rather than in trabecular bone [25], [26], [27]. Consequently, just as either continuous or intermittent endogenous or exogenous PTH may have positive effects on trabecular bone, our data suggest that either continuous PTH1-84 or intermittent PTH1-34 can have positive effects on endochondral bone formation during fracture healing.

Overall therefore our studies demonstrate the important role that endogenous PTH plays in fracture healing and show that exogenous PTH is more efficacious in increasing callus areas, endochondral bone formation and callus remodeling and in producing a bone of greater mechanical strength, in the presence than in the absence of endogenous PTH.
